# Reduced energy availability: implications for bone health in physically active populations

**DOI:** 10.1007/s00394-017-1498-8

**Published:** 2017-07-18

**Authors:** Maria Papageorgiou, Eimear Dolan, Kirsty J. Elliott-Sale, Craig Sale

**Affiliations:** 1 Musculoskeletal Physiology Research Group, Sport, Health and Performance Enhancement Research Centre, School of Science and Technology, Nottingham Trent University, Nottingham, NG11 8NS UK; 2 Applied Physiology and Nutrition Research Group, University of Sao Paulo, São Paulo, Brazil

**Keywords:** Energy availability, Physically active individuals, Female Athlete Triad, Relative Energy Deficiency in Sports, Bone health, Bone metabolic markers

## Abstract

**Purpose:**

The present review critically evaluates existing literature on the effects of short- and long-term low energy availability (EA) on bone metabolism and health in physically active individuals.

**Methods:**

We reviewed the literature on the short-term effects of low EA on markers of bone metabolism and the long-term effects of low EA on outcomes relating to bone health (bone mass, microarchitecture and strength, bone metabolic markers and stress fracture injury risk) in physically active individuals.

**Results:**

Available evidence indicates that short-term low EA may increase markers of bone resorption and decrease markers of bone formation in physically active women. Bone metabolic marker responses to low EA are less well known in physically active men. Cross-sectional studies investigating the effects of long-term low EA suggest that physically active individuals who have low EA present with lower bone mass, altered bone metabolism (favouring bone resorption), reduced bone strength and increased risk for stress fracture injuries.

**Conclusions:**

Reduced EA has a negative influence on bone in both the short- and long-term, and every effort should be made to reduce its occurrence in physically active individuals. Future interventions are needed to explore the effects of long-term reduced EA on bone health outcomes, while short-term low EA studies are also required to give insight into the pathophysiology of bone alterations.

## Introduction

Exercise can be osteogenic, with athletes having higher bone mineral density (BMD), favourable adaptations to bone microarchitecture, particularly at weight-bearing sites, and greater bone strength than their sedentary counterparts [1–4]. Sports that convey high-impact, multi-directional movement patterns and unaccustomed loads, likely convey the greatest benefit to bone [5, 6]. In contrast, participation in sports involving lower-impact, repetitive loading cycles (such as endurance running) or non-weight-bearing sports (such as cycling and swimming) do not typically elicit any exercise-induced skeletal benefits. Cyclists and jockeys present with lower BMD compared to athletes partaking in weight-bearing sports or non-athletic controls [1, 2, 7–9]. Experimental studies have indicated that although exercise per se may not be detrimental to bone, bone may be negatively affected when exercise in undertaken in the presence of energy deficiency [10, 11].

Matching dietary energy intake (DEI) to exercise energy expenditure (EEE), thus maintaining optimal energy availability (EA), is a challenge for many physically active populations, including elite and recreational athletes; individuals in arduous occupational roles (e.g., military recruits) and regular exercisers, particularly those engaged in high-intensity training programs, or those who train for endurance events [12, 13]. Conversely, negative bone health outcomes arising from low EA are well identified in physically active women and are summarised as the Female Athlete Triad [14, 15]. More specifically, female athletes experiencing low EA and/or reproductive disorders are likely to develop low BMD [14] and alterations in bone microarchitecture and bone strength [3, 4] that might increase the risk of osteoporosis and fracture (stress fracture injury or osteoporotic fracture) during or after the cessation of their careers [16, 17]. These findings underpin the long-term and sometimes irreversible effects of low EA on bone health in this population and reinforce the need for optimal nutrition and exercise practices. Recent literature suggests that a number of active populations including male athletes and athletes with disabilities may also experience negative bone health consequences, alongside many other performance and health-related impairments, as a result of low EA [i.e., the Relative Energy Deficiency in Sports (RED-S) model] [18, 19].

Despite the rapidly growing body of literature on the long-term manifestations of reduced EA on bone health, little is known about the sequence of events through which reduced EA leads to these bone outcomes in physically active populations. Bone metabolic activity can be assessed indirectly by determining the levels of bone metabolic markers, which indicate bone formation or bone resorption [20], to provide useful information on short-term dietary and exercise interventions [10, 11, 21]. Alterations in bone metabolism may precede the detection of established changes in bone mass and structure [22]. Insight into potential mechanisms mediating initial bone metabolic responses can also be gained by investigating the short-term effects of low EA on endocrine factors responsive to changes in energy status [e.g., insulin, leptin, triiodothyronine (T_3_), insulin-like growth factor (IGF-1)] and markers of reproductive function [10, 11].

This review discusses the concept of low EA in an exercise physiology/sports nutrition context, along with models that describe the relationship between EA and bone health in physically active individuals, namely the Female Athlete Triad and the RED-S models. The current evidence regarding the short- and long-term effects of reduced EA on bone health in active populations is synthesised, whilst identifying potential mechanisms through which short- and long-term reduced EA may influence bone. Nutritional strategies, which may reduce the negative effects of low EA on bone outcomes, are described. Current knowledge gaps are also identified and directions for future research are provided.

## Energy balance vs. energy availability

The concept of energy balance is often used to explore the effects of insufficient DEI relative to total energy expenditure (TEE) in physically active individuals [23]. This approach is based on the first law of thermodynamics, which states that “energy cannot be created or destroyed, it can only be converted from one form to another” [24]. When considered in relation to human physiology and bioenergetics, this law means that body mass will remain constant if the intake of kilocalories equals that expended. A deficit or surplus of calories will therefore result in body mass gain or loss. This simple equation, energy balance = DEI − TEE, provides a useful construct to consider the overall regulation of human body mass. This approach has practical limitations, particularly when utilised within an exercise physiology context. Both DEI and TEE are complex and interrelated phenomena that are difficult to measure. TEE comprises basal metabolic rate, the energy expended in physical activity (voluntary, i.e., purposeful movement and involuntary, e.g., shivering) and the energy required to ingest and process food for storage and use, termed the thermic effect of food. Numerous factors influence each of these components, including sex, age, training status, nutritional status, body composition and genetics [25], rendering accurate measurement difficult, particularly in the field setting where laboratory equipment (e.g., metabolic chambers and indirect calorimetry devices) is not available. DEI is generally assessed using subjective methods (e.g., food records; dietary recall and food frequency questionnaires). Review papers have been conducted to summarise the relationships between subjectively reported DEI and TEE assessed by doubly labelled water. These investigations reported a mean reporting bias of −20 ± 15% in non-athletic populations [26] and −20 ± 14% in athletes [27]. This reporting bias is thought to originate from a combination of both unintentional (e.g., forgetting to record all foods, or under-estimating portion size) and/or intentional (e.g., desire to provide information that will be viewed positively by the researcher) errors [28].

In addition to the errors associated with measuring the core components of the energy balance equation, this model also assumes that all physiological systems are functioning at an optimal level, which may not necessarily be the case. It has been proposed that an energy-deficient organism may reduce basal metabolism in an attempt to restore balance, albeit with a suppression of non-immediately essential physiological functions [12]. Stubbs et al. [29] studied eight lean men who resided in a metabolic chamber for 7 days in order to assess the effect of no exercise vs. an exercise protocol contributing to EEE of 10.2 kcal kg day^−1^ (achieved through 3 × 40 min cycling sessions at 65.1 ± 9% of *V*O_2_max), along with two dietary manipulations [high fat (50% DEI) vs. low fat (25% DEI)] on compensatory changes in DEI and energy expenditure [29]. Three of these treatments induced a mean negative energy balance throughout the study (i.e., high fat + exercise: −908 kcal day^−1^; low fat/no exercise: −478 kcal day^−1^ and high fat + exercise: −1960 kcal day^−1^). Interestingly, regression of trends in DEI, TEE and energy balance against study days showed a daily decrease in TEE of −76 kcal day^−1^ in the exercise trials, due to a progressive decrease in non-exercise EE. This resulted in a compensatory daily reduction to the extent of negative energy balance experienced by the participants. It was projected that should these compensatory trends continue, energy balance would be restored to zero within 2–4 weeks [29]. These data provide a useful illustration of how energy balance can be achieved in an energy-deficient state, albeit with an accompanying suppression of physiological and metabolic processes [12]. It is important to note, however, that the projections reported by Stubbs et al. (i.e., that energy balance would be restored to zero after 2–4 weeks) assume that the compensatory changes in TEE would continue in a linear direction [29]. This has yet to be directly investigated and studies investigating the long-term response of the body to energy deficiency are required.

In response to the aforementioned limitations of the energy balance equation, the measurement of EA rather than energy balance has become more commonplace in the field of exercise physiology. In the case of athletes, EA is defined as DEI minus EEE (EA = DEI − EEE) normalised for lean body mass (LBM) or fat-free mass. This approach reduces the degree of measurement error, as researchers only need to measure EEE, rather than TEE, which also comprises basic metabolic rate and the thermic effect of food. As such, the potential measurement error associated with DEI and EEE will be less than the cumulative error associated with measuring all components of DEI and TEE. Additionally, the EA concept and equation considers the amount of energy that is available for basic physiological function, and is therefore an input to the system. In contrast, the energy balance equation describes energy as an output of the system, and provides no insight into the state of function of various physiological processes [12].

During the 1990s and early 2000s, studies using the concept of EA in humans provided evidence that impairment of physiological functions previously considered to be due to the “stress” of exercise, were unrelated to exercise per se, but were actually a consequence of reduced EA [30, 31]. Reduced EA at 30 kcal kgLBM^−1^ day^−1^ may be the “threshold” of available energy required for usual reproductive and osteogenic function in sedentary women [10, 31], a value which closely corresponds to resting metabolic rate. It is important to note, however, that these studies reported markers of reproductive function and bone metabolism at discrete EAs. Identification of the specific “threshold” at which function actually declined is, therefore, not possible. In reality, the relationship between EA and physiological function is likely to be subject to a large degree of within and between participant variability [32] (Papageorgiou et al., under review).

## Energy availability and bone health: the Female Athlete Triad and the RED-S models

Two models, the Female Athlete Triad [14, 15] and the RED-S models [18] have been proposed to describe the interrelationships among energy deficiency and bone health in physically active populations. The first published paper in this area described the Female Athlete Triad as the relationship between disordered eating, amenorrhea and osteoporosis in physically active girls and women [33]. In 1997, the American College of Sports Medicine published their first position statement on the Female Athlete Triad to provide evidence-based information about screening, diagnosis, prevention and treatment [34]. Originally, the co-existence of all three components was a pre-requisite for the diagnosis of the Female Athlete Triad, although, a decade later, an updated version of the Female Athlete Triad described the original components of the Triad as a continuum from health to disease [14]. The healthy state is characterised by optimal/adequate EA (45 kcal kgLBM^−1^ day^−1^), eumenorrhea and normal BMD, that is followed by suboptimal EAs (between 30 and 45 kcal kgLBM^−1^ day^−1^), subclinical reproductive disorders (anovulation and luteal phase defects) and low BMD (BMD *Z* score between −1.0 and −2.0 with risk factors for fracture). The clinical manifestations include low EA (<30 kcal kgLBM^−1^ day^−1^) with or without disordered eating, functional hypothalamic amenorrhea and osteoporosis (BMD *Z* score ≤2.0 in the presence of clinical risk factors for fracture). Notably, although the Triad components are believed to be interrelated, the progression of each component in the continuum may occur independently and does not necessitate the presence of the other two. This updated version of the Female Athlete Triad acknowledges the occurrence and consequences of unintentional energy deficits, as indicated by replacing disordered eating with low EA and by recognising that low EA/energy deficiency is an underlying cause of the other two components. In 2014, the Female Athlete Triad Coalition published a consensus statement, which was adopted by the American College of Sports Medicine and the American Bone Health Alliance, with guidelines regarding treatment and management of the components of the Female Athlete Triad and the introduction of an algorithm for return-to-play of athletes with the Triad [15].

In 2014, an International Olympic Committee expert panel extended the concept of the Triad, to the RED-S models, in order to broaden its definition and consequences. Thus, the RED-S model extends the adverse effects of low EA on health and performance beyond those originally described in the Female Athlete Triad [18]. In addition to female athletes, the RED-S model proposes that male athletes, non-Caucasian athletes and athletes with disability may also experience low EA and its subsequent adverse health (including bone health) and performance (including risk of musculoskeletal injuries) consequences [18]. This change in terminology has been the subject of some debate [35, 36]; with concerns expressed regarding the expansion of affected populations due to insufficient evidence and the potential distraction away from female athletes [35]. The addition of a number of health consequences has also been criticised, as it has been suggested that this may oversimplify complex interactions and diverts attention from the well-researched clinical outcomes included in the Female Athlete Triad [35]. Finally, it has been suggested that the RED-S risk assessment and return-to-play models require further research in order to allow more evidence-based quantifiable recommendations to be made (e.g., ranges for some risk factors, the number of risk factors required for return-to-play decisions to be made) [35].

The Female Athlete Triad and RED-S models are consistent with clinical observations in non-athletic individuals with long-term energy deficiency [37]. Studies in adult women and adolescent girls with anorexia nervosa have reported low areal BMD at multiple bone sites [38–41], impairments in parameters of bone architecture (e.g., reductions in cortical thickness, cortical area, trabecular area and trabecular volume) [42, 43], reductions in estimates of bone strength [42] and increased fracture risk [44]. Altered bone metabolism, as suggested by increased markers of bone resorption and decreased markers of bone formation, have also been reported in adult women with anorexia nervosa [44]. Studies in men with anorexia nervosa are limited, but show high rates of low BMD *Z* scores [45, 46] and suppressed bone metabolism (lower bone formation and resorption markers compared to controls) [45].

Research in anorexia nervosa has also provided some insight into the mechanisms that may mediate bone loss in this condition [37]. These include changes in body composition (reductions in LBM and fat mass) [39, 41] alterations in reproductive (oestrogen and androgen deficiency) [39, 45] and energy regulatory [e.g., decreased leptin, insulin, IGF-1, increased cortisol and growth hormone (GH) levels] hormones [41–43, 47, 48]. Differences between non-athletic individuals with eating disorders and active individuals under low EA, do not allow the extrapolation of these findings in the latter. For example, only some athletic individuals develop eating disorders and disordered eating [12]. In addition, the degree of the energy deficit attained by physically active populations is commonly less severe than that attained by patients with anorexia nervosa, while exercise (particularly weight-bearing activities) may counteract some of the unfavourable effects of low EA on bone in physically active persons [49, 50]. The literature in the area of eating disorders is, however, valuable in supporting the existing literature in the area of EA and bone health in physically active individuals [14, 15, 18] and in generating hypotheses related to the bone response to low EA and the potential mechanisms through which bone may be affected in energy-deficient states.

## Bone response to reduced energy availability in active populations

### Short-term effects of reduced energy availability on bone metabolism

A limited number of studies have investigated the short-term effects of energy deficiency (based on energy balance or EA) on bone metabolism in physically active individuals [11, 51] (Papageorgiou et al., under review). An additional two studies have been conducted in normal-weight sedentary populations [10, 21]. Ihle and Loucks [10] explored the effects of three distinct levels of reduced EA, at 30, 20 and 10 kcal kgLBM^−1^ day^−1^, on bone metabolic markers in sedentary, eumenorrheic women, compared to a balanced EA at 45 kcal kgLBM^−1^ day^−1^. All three levels of reduced EA were achieved by a combination of dietary energy restriction (DEI: 45, 35 and 25 kcal kgLBM^−1^ day^−1^) and walking exercise (EEE: 15 kcal kgLBM^−1^ day^−1^) [10]. This study showed a dose–response relationship between reduced EA and bone metabolic marker concentrations. Carboxyl-terminal propeptide of procollagen type 1 (P1CP) and total osteocalcin (both bone formation markers) were significantly reduced by 12 and 11% from baseline at 30 kcal kgLBM^−1^ day^−1^, whereas urinary amino-terminal cross-linked telopeptide of type I collagen (NTX) (a bone resorption marker) significantly increased (+34% from baseline levels) in the severely restricted EA condition at 10 kcal kgLBM^−1^ day^−1^ only [10]. These data were, however, from sedentary women who differ from their physically active counterparts in bone characteristics (e.g., baseline levels of bone metabolic markers and bone strength), body composition and training adaptations [4, 52, 53]. Additionally, the bone metabolic markers used to assess bone formation and resorption [10] are not recommended as reference analytes by the International Osteoporosis Foundation and the International Federation of Clinical Chemistry and Laboratory Medicine [20]. This is mainly due to the errors introduced when bone metabolic markers are determined in urine samples (e.g., requirements for creatinine corrections) and because of the heterogeneity of their fragments [20]. For example, osteocalcin is found in different forms (i.e., carboxylated, under- or decarboxylated) and sizes (i.e., small, medium and large molecules), which may indicate bone formation, bone resorption or may even be more indicative of energy metabolism rather than bone metabolism [54].

In order to examine the impact of reduced EA in physically active individuals, our recent study explored the short-term effects (5 days) of low EA at 15 kcal kgLBM^−1^ day^−1^, attained by diet (DEI: 30 kcal kgLBM^−1^ day^−1^) and exercise (EEE: 15 kcal kgLBM^−1^ day^−1^-running protocol) on bone metabolic markers in physically active women (Papageorgiou et al., under review). Low EA resulted in a significantly higher β-carboxyl-terminal cross-linked telopeptide of type I collagen (β-CTX) (reference marker of bone resorption) area under the curve (AUC) and a significantly lower amino-terminal propeptide of procollagen type 1 (P1NP) (reference marker of bone formation) AUC compared to a controlled balanced EA at 45 kcal kgLBM^−1^ day^−1^. The findings of this study confirm that altered bone metabolism, favouring resorption, following low EA is not confined to sedentary women [10], but is also evident in their physically active counterparts. Some changes in hormones known to respond rapidly to states of energy deficiency, including reductions in insulin and leptin, were also reported in response to low EA, which is consistent with other short-term experiments [10]. The actions of insulin and leptin on bone have been previously characterised and insulin receptors are present on both osteoblasts and osteoclasts [55, 56]. In vivo, insulin increases bone formation [57] and decreases bone resorption [58]. Leptin may exert its bone effects directly through its receptors on osteoblasts and chondrocytes, but also, indirectly, by altering other hormones including oestrogen, cortisol, IGF-1 and PTH that may, in turn, mediate bone responses—for a review see [59]. No changes in 17β-oestradiol concentrations (a marker of reproductive function) were reported in response to short-term reduced EA in physically active women (Papageorgiou et al., under review), which contradicts previous work in sedentary women that has shown reductions in oestrogen concentrations [10] and reductions in luteinising hormone pulsatility [31]. This could be due to the short timeframe and/or the solitary measurement of 17β-oestradiol levels in Papageorgiou et al. (under review) versus the 24-h pooled analysis conducted by Ihle and Loucks [10].

Men may be more resistant to the negative effects of short-term low EA than women, due to factors such as the reduced energy cost of reproduction or a bone protective influence of androgens [35]. In order to investigate this further, the same experimental protocol described above (Papageourgiou et al., under review) was conducted in a group of physically active men. In contrast to the findings reported in women, there were no significant alterations to either P1NP or β-CTX AUCs. High inter-individual variability in β-CTX and P1NP responses were, however, reported, indicating that low EA affected some, but not all men. Insulin, leptin or T_3_ were not affected, supporting the overall absence of significant bone metabolic marker results. Zanker and Swaine [11] demonstrated that energy restriction through exercise (60 min running) and a 50% restriction in estimated dietary energy requirements reduced P1NP levels in parallel with IGF-1 levels, but did not alter urinary NTX in trained men [11].

In a preliminary, direct sex comparison of bone metabolic marker responses to low EA, no significant differences in bone metabolism were shown between active women and men; women—β-CTX: +19%, P1NP: −13%; men—β-CTX: +12%, P1NP: −14% (Papageorgiou et al., under review). Responses of hormones [insulin, leptin, T3, IGF-1 and glucagon-like peptide 2 (GLP-2)] to low EA did not vary between sexes, supporting the absence of sex differences in bone metabolic responses, which is in line with some [60], but not all previous studies [61, 62]. Low EA alters reproductive hormones (e.g., suppressed luteinising hormone pulsatility, reduced oestrogen levels) [31] and contributes to the development of reproductive disorders in women [32]. Future studies are needed to investigate the effects of low EA on markers of reproductive function, particularly in men, and their impact on bone metabolism. Studies to compare the contribution of markers of reproductive function to bone metabolism in response to low EA in men and women are also required, which will contribute to the RED-S paradigm, by integrating a direct sex comparison.

### Long-term effects of reduced EA on bone health

No controlled, longitudinal or intervention-based studies have directly examined the response of bone to long-term reductions in EA. A number of cross-sectional studies are available that report data related to bone health and EA in track and field athletes [63], endurance runners [64, 65] and high-school varsity athletes competing in a variety of sports [66]. These studies have primarily reported the frequency of Female Athlete Triad component occurrences and have concluded that 71 ± 27% of participants had reduced EA (<45 kcal kgLBM^−1^ day^−1^), 45 ± 16% had menstrual dysfunction and 34 ± 33% had low BMD (*Z* score <−1). The aforementioned studies represent a wide range of athletes, and athletes from some sports may be more susceptible to the Triad components than others. For example, the prevalence study by Goodwin et al. [64] was conducted using elite female Kenyan long and middle distance runners, and reported reduced EA (<45 kcal kgLBM^−1^ day^−1^) in 92% of the participants. In support of this, a systematic review investigating the individual and combined prevalence of the Female Athlete Triad in different groups reported that athletes competing in lean sports, i.e., “sports that place an emphasis on endurance training, low body weight, lean physique and aestheticism”, were more likely to exhibit one or more components of the Triad [67]. The authors of this review did, however, report that variations in study design and methodological limitations, including the difficulty of measuring the Triad components in a field setting, limited interpretation of the results attained. It is important to consider that the studies described here [63–67] were designed to assess prevalence of the Triad components and did not directly assess the relationship between low EA and bone health in active populations. Inferences related to the direct relationship between these components cannot, therefore be made.

Cross-sectional studies have provided some indirect insight into the long-term influence of energy deficiency on parameters of bone structure and function [8, 9, 68–71]. Taken collectively, these studies support the hypotheses generated from the short-term studies described in “Short-term effects of reduced energy availability on bone metabolism”, and indicate that bone metabolism and structure is negatively affected by prolonged exposure to an apparently insufficient DEI in relation to TEE in active individuals. De Souza et al. [68] evaluated the independent and combined effect of oestrogen and energy deficiency [defined as resting energy expenditure (REE) ≤90% of predicted REE according to the Harris–Benedict equation] on bone metabolism in premenopausal exercising women. This study reported that energy deficiency had an independent and negative impact on bone metabolism, as evidenced by a suppression of osteocalcin in the energy-deficient group, while outcome variables related to REE were significant predictors of PINP and urinary CTX levels [68]. More recently, the same group conducted a similar study to investigate the independent and combined influence of energy and oestrogen deficiency on bone, as assessed by peripheral quantitative computed tomography (pQCT), and showed that energy deficiency independently affected bone, with a main effect analysis reporting a relationship between energy status and tibial volumetric BMD, geometry and estimated bone strength [69]. Zanker and Swaine [71] examined the relationship between bone metabolic markers, indices of nutritional status and indicators of reproductive function in female distance runners. Evidence of down-regulated bone formation (reduced bone-specific alkaline phosphatase and osteocalcin) was prevalent among the group of energy-deficient individuals who were also experiencing menstrual irregularities (amenorrhea or oligomenorrhea) [71]. Similarly, evidence from a series of studies on male horse-racing jockeys have shown that long-term exposure to reduced EA may negatively impact bone health in this group. Investigations have reported chronic energy restriction in an attempt to “make-weight” for racing [72, 73] and EA below 30 kcal kgLBM^−1^ day^−1^ in male horse-racing jockeys [74]. Additionally, reduced bone mass [75], reduced cortical area and tibial strength strain index [76] and increased urinary NTX/creatinine [8] have been reported in jockeys, which were attributed to a chronic state of energy deficiency in this group [77]. Wilson et al. [70] reported lower BMD in male jockeys than in female jockeys, suggesting that the bones of male jockeys may be more susceptible to the negative effects of weight-making practices than their female counterparts. It is possible that the results reported within the study by Wilson et al. [70] were due to a reduced frequency of weight-making practices in female jockeys. An alternative explanation, however, relates to a protective influence of adipose tissue mass on bone in females when compared to males [78]. This may be mediated through the effect of oestrogen, which is involved in the regulation of bone homeostasis [79] and is present in higher quantities in women compared to men. Adipose mass (which was reported to be higher in the female jockeys under investigation in the study by Wilson et al. [70]) is a key source of aromatase, which contributes to oestrogen synthesis from androgen precursors [80], and may have influenced the results attained [70]. This hypothesis is speculative, however, and controlled studies investigating a potential sexual dimorphism in the response of bone to chronically reduced EA are warranted.

Reduced EA exerts a direct effect on bone [68, 69]. It may also indirectly influence bone, through suppression of the hypothalamic–pituitary–ovarian axis, with subsequent reduction of oestrogen levels and associated reproductive dysfunction. In order to investigate this, a number of studies have been conducted on athletes experiencing menstrual dysfunction, which have shown reduced bone mass [81–84], negative effects on bone microarchitecture [3, 4, 85] and an increase in stress fracture incidence [17] in amenorrheic or oligomenorrheic athletes, when compared to their eumenorrheic counterparts. These findings were largely attributed to a probable negative energy balance. Indeed, the negative bone effects reported in the energy-deficient groups in the studies by De Souza et al. and Southmayd et al. [68, 69] were exacerbated in the group who were defined as being both energy and oestrogen deficient. Thus, Southmayd et al. [69] suggested that the combination of energy and oestrogen deficiency is more detrimental to bone than energy deficiency alone.

Some studies have reported biochemical data in order to provide insight into the mechanistic pathways through which reduced EA may impact bone. Collectively, these findings provide evidence of energy conservation in apparently energy-deficient athletes [8, 68]. For example, increased sex hormone-binding globulin, with a subsequent reduction in bioavailable testosterone, and decreased IGF-1 were reported as independent predictors of bone mass in a group of male horse-racing jockeys [8], indicating a down-regulation of anabolic processes in this group. Similarly, De Souza et al. [68] reported indirect biochemical evidence of energy conservation in a group of energy and oestrogen-deficient women, as evidenced by reduced T_3_ and increased ghrelin levels. Kaufman et al. [86] reported that resting metabolic rate was predictive of BMD in a group of elite professional ballet dancers. The authors suggested that the reported correlations between resting metabolic rate and BMD may be due to the nutritional habits of the dancers, a group who have previously been reported to routinely employ energy restricted diets, and to be prone to development of eating disorders, in an attempt to maintain the lean physique favoured in this aesthetic sport [87]. Kaufman et al. [86] suggested that the low BMD reported in their study may be caused, at least in part, by a suppression of resting metabolic rate in response to a negative energy balance. This suggestion is, however, speculative, since this cross-sectional study was not designed to directly investigate the relationship between energy balance and BMD in this group. Taken collectively the studies described within this section, support the theory that the body responds to reduced EA by down regulating basic physiological and anabolic processes. Further well-designed and controlled trials are required to confirm the mechanistic pathways through which this occurs.

An important factor to consider when evaluating the impact of reduced EA on bone health is the potential influence of sport type. Many of the studies described within this review were conducted using athletes who participate in sports that provide no-to-low impact and repetitive loading cycles, thus providing a low osteogenic stimulus [9, 64, 65, 71, 88, 89]. Conversely, sports that convey high-impact, multi-directional movements and unaccustomed loads are thought to provide a higher osteogenic stimulus [88] and there is some evidence to suggest that the mechanical loading afforded by particular sports may offer protection to bone under conditions of reduced EA. For example, participation in weight category sports may theoretically pose a risk to bone. Weight-category athletes generally strive to compete in the lowest weight class possible, as this is widely believed to convey a competitive advantage [90]. This is often achieved by severely restricting DEI [91], which may have consequences for bone, as described throughout this review. This notion was investigated by Dolan et al. [9] who compared groups of age, gender and body mass index (BMI) matched weight-category athletes (elite amateur boxers and horse-racing jockeys) and reported higher total body, lumbar spine and femoral neck bone mass in boxers, compared to flat and national hunt jockeys. Regression analysis showed height and LBM to be the primary predictors of these differences. A group-specific factor was, however, also identified as influential and this was hypothesised to relate to the degree of mechanical loading experienced by both groups [9]. This hypothesis is further supported by the results of Trutschnigg et al. [92] who reported high bone mass in paper (46 kg) to middle weight (70 kg) female boxers when compared to active controls, despite a high prevalence of oligomenorrhea and low BMI in the female boxers. Similar results have been reported in gymnasts, a group frequently reported to have low EA [93, 94] and high incidence of menstrual dysfunction [50, 94, 95]. Gymnasts have also been reported to have higher BMD than non-gymnasts [5, 6, 50], a finding which is often attributed to the high-impact nature of the sport. Caution must be taken when interpreting these results, since BMD is not necessarily a good indicator of bone strength in athletic populations, who may have higher strength requirements than the general population for whom the reference-based normative values have been developed. Indeed, a recent paper by Tenforde et al. [96] reported that a high proportion of gymnasts were classified as “moderate–high risk” according to the Female Athlete Triad Cumulative Risk Assessment, and were subsequently more likely to sustain a stress fracture injury.

The available research indicates that bone is negatively affected by long-term exposure to reduced EA, although factors such as reproductive function and sport-type may mediate this response. It is important to note, however, that the majority of studies described within this section were not specifically designed to evaluate the influence of reduced EA on bone health, and, as such, the conclusions drawn from these investigations are indicative, rather than definitive. As identified in “Energy balance vs. energy availability”, both DEI and energy expenditure (EEE or TEE) are difficult to measure accurately, and many of the available studies have used imperfect assessment modalities (e.g., self-reported food records). Chronic exposure to insufficient energy intake is often accompanied by macro- and micro-nutrient deficiencies, making it difficult to isolate the influence of reduced EA per se. The indirect evidence described within this section supports the hypothesis that the body may downregulate aspects of osteogenic function during times of reduced EA, in an attempt to conserve energy for more immediately essential functions. Well-designed and robustly controlled trials are required to confirm these theories and to more fully evaluate the mechanistic pathways through which chronic exposure to low EA may impact bone structure, function and metabolism.

## Nutritional strategies to protect bone during periods of reduced EA

Currently limited research is available that directly investigates the influence of nutritional strategies on bone during periods of reduced EA in active individuals. Indirect evidence does, however, support the adoption of strategies that might protect bone in athletes with high levels of EEE and/or low EA. Ensuring EAs of 30–45 kcal kgLBM^−1^ day^−1^ is the first and most obvious step. This may be logistically difficult, given the heavy training schedules of many athletes, particularly endurance athletes. As such, appropriate DEI should be encouraged and carefully planned by qualified professionals during scheduled periods of high training volume. It is important to note, that EEE does not induce a sufficient compensatory increase in dietary energy intake [29, 97, 98], a finding that might be mediated by a transitory exercise-induced increase in anorexigenic hormones including peptide YY (PYY), pancreatic polypeptide (PP) and glucagon-like peptide 1 (GLP-1) [98]. This is in line with increased PP [99] and PYY [100] previously shown in individuals with anorexia nervosa as the result of long-term energy deficiency. Athletes must, therefore, learn to eat by discipline and not rely solely on physiological queues, such as hunger. Additionally, the available literature reports that disordered eating, and cognitive dietary restraint, are predictive of reduced bone mass in active individuals [81, 101]. Educational interventions that highlight the performance and health-related consequences of reduced EA, along with practical strategies intended to facilitate the athlete to overcome these challenges may be beneficial.

In certain situations, weight loss, or maintenance of a very low body mass, may be a real or perceived contributor toward competitive success and athletes may consciously choose to maintain a state of reduced EA. In this situation, strategies are available that might alleviate the negative osteogenic consequences of reduced EA. LBM has been reported to be the strongest independent predictor of bone mass in a variety of populations [78, 102] and preservation of muscle mass should be prioritised during times of reduced EA. Although research is ongoing, and far from conclusive, maintaining or relatively increasing protein intake has been suggested to be advantageous in preserving LBM during periods of energy deficiency, with intakes of 1.8–2.0 g kcal day^−1^ being recommended [103]. Theoretically, higher animal protein intakes could have a negative impact on bone, through increasing blood acidosis [104], which may subsequently increase osteocyte activity and bone resorption [105, 106], as calcium may be released to buffer the increased acid load. That said, this might not be such an issue where calcium levels are adequate and so higher protein intakes should be accompanied by an adequate intake of calcium [107]. This assertion is supported by data from Josse et al. [108], who reported preservation of LBM and a more positive bone metabolic profile (PINP:CTX ratio) in a group of overweight individuals who were fed a hypocaloric diet, composed of high protein and dairy foods, during a period of exercise and diet-induced weight loss. This indicates that a high-protein and high-dairy intake may be beneficial to bone during times of negative energy balance, although this study was not designed to evaluate the independent influence of these strategies.

Increasing meal frequency can facilitate a reduction of within-day variability in DEI and may be particularly beneficial to athletes whose intensive training schedules render it difficult to consume sufficient caloric and nutrient intake during more traditional set meal-times [109]. This strategy has been reported to suppress LBM loss in a group of boxers during a period of dietary restriction [110], which may indirectly protect bone. In further support of this, food fractionation, through the provision of four small meals per day, as opposed to a single meal, was reported to augment bone mass and morphology in rats [111], potentially through reducing the extent of diurnal variation in bone resorption. Further controlled trials are required to more conclusively establish the efficacy of this dietary approach, particularly its impact on bone health during periods of reduced EA.

Energy deficiency is often accompanied by deficits in essential micronutrients [112, 113], which may independently impact bone health. No research is available that directly investigates the independent versus combined influence of nutrient and caloric deficiencies on bone in active individuals. Many micronutrients are known to directly impact bone health (e.g., calcium, vitamin D and magnesium) and therefore adequate intake is essential [114]. Additionally, other nutritional elements, such as anti-oxidants, may indirectly impact bone health through their ability to reduce oxidised particles. Oxidised particles are important metabolic regulators and have been reported to increase osteoclast differentiation and therefore, bone resorption [115, 116]. Exercise-induced oxidised particles may act as vital signalling molecules to mediate the bone-adaptive response to exercise. Redox balance is, however, dependent on the ability of the body’s anti-oxidant system (including exogenous anti-oxidants such as vitamins A, C, E, beta carotene and selenium) to reduce oxidised particles. Chronic redox imbalance and increased exposure to oxidative stress has been implicated in the development of a range of conditions, including osteoporosis [117, 118]. It is important, therefore, that the micronutrient adequacy of the diet is preserved during periods of intentional or unintentional reduced EA and this can be achieved by consuming a wide variety of nutrient-dense foods; e.g., fruit and vegetables; whole grains; high-quality protein including dairy, fish, poultry, meat, legumes and nuts.

## Future research

Research regarding the implications of reduced EA on bone health in active individuals has been conducted for over 30 years and important advances have been made to our understanding of these interrelated phenomena during this time. These include the recognition of EA as the underlying factor for compromised bone health and reproductive function [14, 15, 18], the expansion of the spectrum of negative consequences arising from low EA and the addition of affected active populations [18]. There are, however, several knowledge gaps remaining on the short- and long-term effects of reduced EA on bone health.

Thus far, only a limited number of short-term, intervention studies have been conducted on EA and bone metabolism in physically active individuals, making it difficult to draw firm conclusions. Low EA (15 kcal kgLBM^−1^ day^−1^) may induce an increase in bone resorption with a synchronous decrease in bone formation in physically active women (Papageorgiou et al., under review), whereas energy deficiency in physically active men has resulted in conflicting findings, such as reduced bone formation [11] or no changes in bone metabolism (Papageorgiou et al., under review). Thus, future studies should explore if a more severe level of reduced EA (<15 kcal kgLBM^−1^ day^−1^) or a longer timeframe would elicit consistent bone metabolic responses in physically active men.

It remains uncertain whether short-term, low EA, achieved via diet or exercise, has the same effects on bone metabolism. Current studies have examined dietary restrictions alone [21] or in combination with exercise [10, 11] (Papageorgiou et al., under review) on bone metabolic markers, but no study has yet explored the same level of reduced EA by diet or exercise independently, which is a critical question ahead of determining prevention and treatment strategies. Existing studies have used walking [10] or running [11] (Papageorgiou et al., under review) to manipulate EEE. Weight-bearing exercise provides an anabolic stimulus to bone [88], which may have masked some of the effects of low EA on bone metabolism. Exploring the effects of low EA on bone metabolism using non-weight-bearing exercise protocols will be useful in clarifying if the type of exercise influences the response of bone to reduced EA.

Current knowledge on the long-term effects of energy deficiency/low EA on parameters of bone health comes from cross-sectional studies [8, 9, 68, 69]. Prospective, longitudinal, well-controlled studies are required to more fully evaluate bone mass, structure and metabolism and the mechanistic pathways through which long-term exposure to low EA may impact bone. The available evidence for men is more limited than for women and there is need for future research to determine male-specific meaningful ranges and cut-offs for optimal and low EA related to clinical outcomes. The vast majority of existing research has been conducted in Caucasian, active individuals, but not in other populations proposed by the RED-S models, namely athletes with different racial backgrounds and athletes with disabilities, underpinning the need for future research to explore the effects of short-term and long-term low EA on bone metabolism and health in these physically active populations.

## Summary and conclusions

This review has summarised the available evidence regarding the influence of short- and long-term reduced EA on bone in physically active populations. Low EA may induce an increase in bone resorption that is accompanied by a concomitant decrease in bone formation in physically active (Papageorgiou et al., under review) and sedentary [10] women. Bone metabolic marker responses to low EA are less consistent in physically active men, as evidenced by the equivocal findings from available studies and higher inter-individual variability [11] (Papageorgiou et al., under review).

Findings from cross-sectional studies conducted on groups of participants who have experienced a longer-term exposure to reduced EA supports the theory that bone is affected by low EA, as evidenced by lower bone mass and structure, and an altered bone metabolic profile in energy-deficient individuals, when compared to their energy-replete counterparts [3, 4, 8, 68, 69]. Factors such as reproductive function and sport-type may at least partially mediate this response, with reproductive dysfunction appearing to exacerbate the negative influence of reduced EA [68, 69]. Conversely, participation in sports that convey a high level of mechanical loading may attenuate the negative influence of reduced EA, although further research is required to test this hypothesis. Taken collectively, the available evidence suggests that reduced EA has a negative influence on bone, and every effort should be made to reduce its occurrence in physically active individuals.
